# Continuous heart rate variability monitoring, stress and recovery in doctors: a systematic review and meta-analysis

**DOI:** 10.1093/occmed/kqaf101

**Published:** 2025-10-28

**Authors:** L Kane, D Powell, K R Martin, C Rees, J Curran, D Ball

**Affiliations:** Academic Primary Care and Health Psychology, Institute of Applied Health Sciences, School of Medicine, Medical Sciences and Nutrition, University of Aberdeen; Emergency Department, NHS Grampian; Academic Primary Care and Health Psychology, Institute of Applied Health Sciences, School of Medicine, Medical Sciences and Nutrition, University of Aberdeen; Academic Primary Care and Health Psychology, Institute of Applied Health Sciences, School of Medicine, Medical Sciences and Nutrition, University of Aberdeen; Emergency Department, NHS Grampian; Emergency Department, NHS Grampian; Sports Science, Institute of Education in Healthcare and Medical Sciences, School of Medicine, Medical Sciences and Nutrition, University of Aberdeen

## Abstract

**Background:**

Burnout is a rising concern among doctors. Heart rate variability (HRV), a non-invasive measure of autonomic nervous system activity, can reflect physiological states of sympathetic (stress) and parasympathetic (recovery) nervous system activity.

**Aims:**

This review aims to evaluate how continuous ambulatory HRV monitoring has been used to understand patterns of stress and recovery in doctors.

**Methods:**

The study protocol was preregistered (PROSPERO CRD42023413282). A comprehensive search was conducted. Studies were eligible if they reported at least one HRV parameter combined with at least one contextual or psychological assessment over a 24-hour period in a doctor population. Methodological quality was assessed using the Joanna Briggs Institute risk of bias assessment for case reports and the Standard for Reporting Diagnostic Accuracy in HRV studies STARD_HRV_.

**Results:**

We identified 805 records of which seven studies met the inclusion criteria. Meta-analysis was carried out for five different HRV parameters. Studies were conducted in seven different countries, ranged in participant numbers from 12 to 54 and each used a different HRV measurement device. There was a statistically significant difference in HRV between stress and recovery periods with four of these five parameters; root mean square of successive differences SMD = −0.63, *P *= 0.005, standard deviation of the NN (inter-beat) intervals SMD = −1.05, *P *= 0.001, low frequency (LF) Standard Mean Difference (SMD) = 0.54, *P *= 0.01, LF/high frequency (HF) SMD = 0.69, *P *= 0.006. The quality of studies was moderate at best.

**Conclusions:**

Continuous HRV monitoring may offer a viable method for tracking stress and recovery patterns that may contribute to burnout.

Key learning points **What is already known about this subject:**

- Urgent solutions for managing occupational stress, burnout and workforce retention in healthcare are required.

**What this study adds:**

- There is international interest in using HRV as a biomarker of stress and recovery in doctors. The quality of existing studies is moderate with high levels of methodological heterogeneity.
- Advancing technology in wearables may provide useful tools for occupational assessments.
- This review demonstrates that several HRV parameters may be used to detect statistically significant changes between stress and recovery.

**What impact this may have on practice or policy:**

- This review suggests that there may be potential to use HRV as a non-invasive biomarker of occupational stress offering occupational health professionals an objective tool to detect and monitor stress related responses in doctors.
- Further high-quality work in the area is required, particularly in a UK medical workforce to further inform the clinical application of HRV as a biomarker for occupational stress monitoring. Such evidence could inform occupational medicine strategies for surveillance, risk assessment and targeted support.

## INTRODUCTION

Burnout is a growing concern among doctors as indicated by the 2023 UK General Medical Council (GMC) survey of over 45 000 doctors [1]. The findings reveal that 66% of trainee doctors and 52% of trainers (assumed to be senior doctors) report moderate to high levels of burnout. Since initiating the survey in 2019, the reported rates of burnout have consistently risen. This indicates a growing problem in the UK doctor population. Notably 80% of Emergency Medicine and Stroke Medicine trainees report moderate to high levels of burnout, while numerous surgical and medical specialities surpass 60% [1]. The training process itself does not seem to be the sole problem, with senior doctors continuing to experience moderate to severe burnout at rates exceeding 50% [1]. This is a particular concern as it takes over 10 years to train doctors to be a General Practitioner (GP) or consultant at a cost of over £500 000 each [2], these senior doctors are responsible for leading clinical service provision, carry significant management responsibilities, are responsible for training and have a wealth of clinical experience that allows them to manage the most complex clinical cases. They cannot be easily replaced. In 2022, 52% of consultant vacancies in England and Wales were unfilled and 14% of senior doctor posts in Scotland were unfilled. This highlights the need to urgently identify and address factors contributing to staff attrition. The reasons behind high levels of attrition will be multifactorial and complex, however the physical and mental health consequences related to burnout will be a contributing factor.

Burnout is characterized as a response to unmanaged chronic workplace stressors [3]. Although some stress can enhance performance, an imbalance between available resources and demands can lead to chronic stress, contributing to burnout [4]. The main resources necessary for job performance include physical equipment, time, mental capacity, and adequate training amongst others. Stressors experienced by individuals accumulate over time, necessitating appropriate recovery in order to prevent chronic stress states. While subjective measures are available to assess the need for recovery [5], little is known about the overall objective stress burden and recovery requirements of doctors working in the UK.

One promising more objective measure may be heart rate variability (HRV). HRV is derived from beat-to-beat variations in heart rate [6]. This is controlled by the primitive autonomic nervous system. The sympathetic branch of the autonomic nervous system, associated with acute stress responses, causes a decrease in HRV, and the parasympathetic branch, associated with rest and recovery, increases HRV. HRV measurements are increasingly employed in sports science as a measure of athlete stress and recovery and are accessible through commercially available wearable devices like WHOOP! ^®^, Garmin^®^, Fitbit^®^, Oura^®^ and Apple Watch^®^. These devices use HRV measurements to indicate various forms of stress, recovery and readiness scores which are user friendly and are increasingly being utilised for managing overall wellbeing in the general population. However, the use of HRV in this way is also controversial in that it is far from the simplistic approach that the algorithms derived within wearable technologies portray [7].

There are several numerical parameters that are derived from HRV measurements, with some reflecting sympathetic nervous system function, some reflecting parasympathetic function, and others reflecting overall balance of the autonomic nervous system function and circadian rhythm. A summary of HRV parameters and their corresponding autonomic nervous system function is provided in Appendix S1. These parameters are significantly more complicated to interpret than commercially derived scores; however, given that HRV can be measured non-invasively and without significant cost, it has been proposed that HRV could be a useful biomarker of occupational stress and recovery in doctors [8,9].

Solely detecting physiological stress is problematic. Normal physiological stress responses are expected, and daily variations are essential for survival [10]. It is for this reason that any ambulatory HRV measurement necessitates an accompanying contextual or psychological measure. Otherwise, it is not possible to distinguish, from the data, healthy physiological responses from those that may be more harmful. What is less well understood is whether HRV can be used to track overall stress burden and recovery requirements that may lead to maladaptive responses, burnout and the associated pathologies such as cardiovascular disease in men and musculoskeletal disease in women [11,12].

This review aims to evaluate the use of continuous ambulatory HRV monitoring, combined with contextual and subjective stress measures, to understand patterns of stress and recovery in medical doctors.

## METHODS

The study protocol was preregistered with PROSPERO prior to commencement: (CRD42023413282) [13].

This systematic review was performed following the guidelines of the Cochrane Handbook of Systematic Reviews of Interventions [14] and the preferred reporting items for systematic review and meta-analysis (PRISMA) statement. Appendix S2 details the PRISMA checklist. Ethical approval was not required.

Studies investigating the use of HRV as a measure of stress in the normal workplace of medical doctors were included. They were required to report at least one HRV parameter, have a minimum of 10 participants, include measurements of at least 24 hours and include at least one contextual or psychological measure alongside HRV (see Table 1). Studies were excluded if they were conducted with medical students as the main study population or if the HRV monitoring period was carried out in simulated or educational environments.

**Table 1.** Inclusion and exclusion criteria using the participants, intervention, comparisons and outcomes (PICO) framework [14]

	Inclusion criteria	Exclusion criteria
Study design	All study types	Books, commentaries, editorials, guidelines, letters, news and opinions, reports, and reviews
Participants	Minimum of 10 participants	
	Medical doctors of all grades and specialties	Medical students
Intervention/exposure	Time spent within a stressful environment in comparison to resting, recovery or less stressful states	No defined stressful environment
		No defined recovery or resting state
Comparisons	Any contextual or psychological measure of stress and/or recovery	No comparison
Outcomes	24 hours or more of HRV monitoring	Less than 24 hours of HRV

The literature search strategy was developed in conjunction with a medical librarian. The searches were conducted in June 2023 and repeated in July 2024, on MEDLINE, the Cochrane Library, Embase, PsychINFO. Reference lists were hand-searched for relevant citations and grey literature was searched in Google Scholar, MedArXiv and PsyArXiv. Studies identified from 1982 to the present day were included.

The search strategy included MeSH and text search terms reflecting the population of interest (doctors). Details of the full search strategy are listed in Appendix S3.

Titles and abstracts retrieved from the search were imported into Covidence (https://covidence.org) and duplicates were automatically removed. Titles and abstracts were reviewed independently by two researchers (L.K. and C.R.) against inclusion criteria. Disagreements were resolved by a third independent researcher (J.C.). Two researchers (L.K. and C.R.) reviewed the full texts against inclusion criteria with a third (J.C.) again resolving any disputes, ensuring that no relevant papers were discarded [14]. Two texts published in a language other than English were translated before screening.

Data were extracted following the predefined criteria outlined in the registered protocol [13]. This included the number of participants, medical speciality, years of clinical experience, length of HRV measurement period, HRV device used, defined stress and recovery periods, HRV parameters reported, STARD_HRV_ [15] criteria and Joanna Briggs Institute (JBI) risk of bias for case series information [16]. Custom data extraction forms were developed in Covidence Data extraction was completed by a single reviewer (L.K.) and double-checked by a second (C.R.).

At the time of registering the review protocol, it was predicted that due to the novel area of study, broad search criteria and predicted heterogeneity, that data synthesis would not be possible. However, following data extraction it became apparent that it would be possible to perform a meta-analysis of five individual HRV parameters. Quantitative analysis was performed using RevMan™ (Web version 5.6.0, The Cochrane Collaboration) random effects analysis [17]. Results are expressed as standard mean difference using Hedges’ adjusted g algorithms, with 95% confidence intervals (CIs). A *P*-value of <0.05 was considered statistically significant. Random effects meta-analysis models were used to account for data heterogeneity. Heterogeneity between studies was assessed by the *I*^2^ score.

All included studies met the Oxford Journal of Epidemiology definition of a case series [18] therefore the JBI case series check list [16] was used to assess overall study quality. Additionally, STARD_HRV_ [15] was used to specifically assess how HRV was recorded and measured during the studies.

## RESULTS

**Figure 1.**
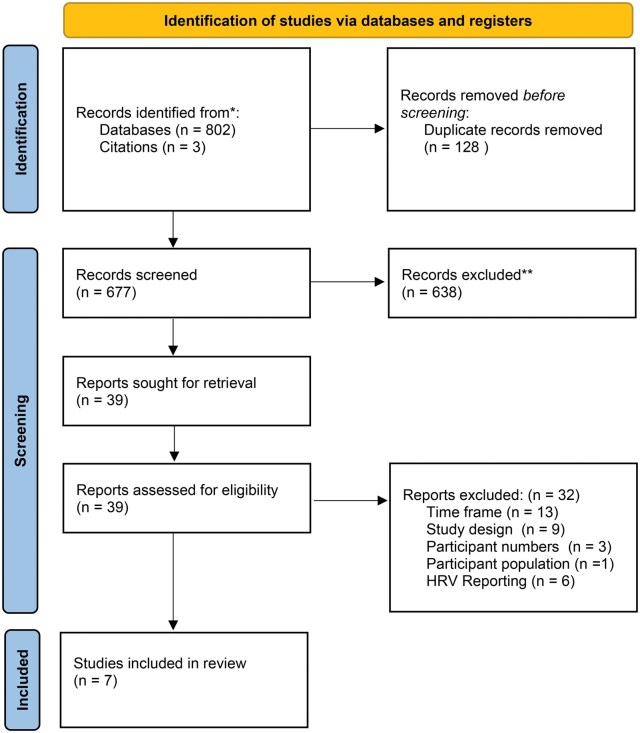
Flowchart according to preferred reporting items for systematic reviews and meta-analysis (PRISMA) methodology.

The search strategy identified 805 results. After duplicate removal and independent review of titles and abstracts, 39 full-text studies underwent review. Seven studies met the inclusion criteria for systematic review [19−25] (Figure 1).

Studies were conducted in a variety of international locations: The Netherlands, Switzerland, Taiwan, Germany, Austria, Denmark, and Mexico. There was a total of 176 participants included spanning a variety of medical specialities. Table 2 describes the included study characteristics. The described high stress and low stress (recovery) periods varied from study to study due to predictable variations in working environment, clinical demands and shifts. High stress periods included operating time, prehospital emergencies, labour ward, and double work duties while the periods of recovery ranged from complete non-working rest to non-clinical administrative work to being completely outside of work. Generally, these were determined or assessed via self-report measures. A different HRV monitor was used in every study, including commercially available devices, medical devices and experimental devices. All studies measured HRV for 24 or 48 hours.

**Table 2.** Characteristics of included studies

Lead author and year of publication	Country	Number of participants	Medical specialty	Stressful environment	Recovery or low stress environment	Contextual or psychological measure	HRV device used	Length of measurement (hours)	HRV parameters reported
Rauchenzauner [24] (2009)	Austria	30	general internal medicine, neurology, ear nose and throat	Nightshifts	Non-working control night	Log-book	CardioMem	24	LF power nu; HF power nu; LF/HF
Hernandez-Gaytan [23] (2013)	Mexico	54	Various	Self-reported low job strain	Self-reported high job strain	Diary	SG-HP3VER306 mode	24	SDNN; LF power ms2; HF power ms2; LF/HF
Martinez de Tejada [19] (2013)	Switzerland	18	Obstetrics and Gynaecology	Labour Ward	Outside work	VAS	Suunto	24	SDNN; RMSSD; LF power nu; HF power nu; LF/HF
Amirian [20] (2014)	Denmark	29	Surgery	On call	Pre-call	Work-plan	Medilog AR12	48	LF power ms2; HF power ms2
Lee [25] (2015)	Taiwan	12	Internal medicine	Double duty	Off duty	Event diary	DR200/Holter	24	SDNN; pNN50; RMSSD; LF power nu; HF power nu
Schneider [26] (2017)	Germany	13	Pre-hospital emergency medicine	Pre-hospital emergencies	Before + after emergency	Time of emergency alarm	Zephyr	24	SDNN; pNN50; RMSSD; LF power nu; LF power %; HF power nu; HF power %; LF/HF
Weenk [22] (2018)	The Netherlands	20	Surgery	Surgery	Baseline/admin work/clinic duties	STAI	Health patch	48	SDNN; RMSSD; LF/HF

No single common HRV parameter was reported to make an overall meta-analysis appropriate. Therefore, subgroup analyses were conducted by grouping studies together based on which HRV parameters they reported for both stress and recovery periods: three studies reported root mean square of successive differences (RMSSD) [19,22,26], four studies reported standard deviation of the NN (inter-beat) intervals (SDNN) [19,22,23,26], three reported high frequency (HF) parameters, three reported low frequency (LF) parameters [19,22,26] and two reported HF LF ratio [22,23].

**Figure 2.**
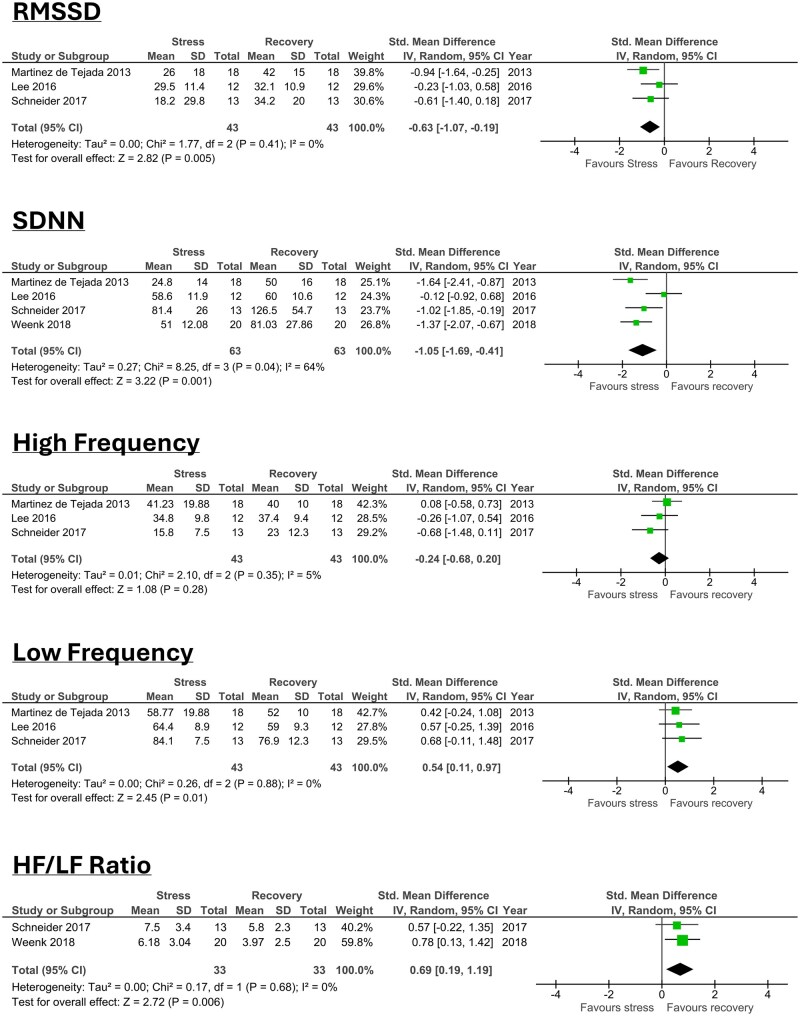
Random-effects meta-analysis assessing the root mean squared of successive differences (RMSSD), standard deviation in N-N interval (SDNN), high frequency (HF), low frequency (LF), and high frequency low frequency ratio (HF/LF) heart rate variability (HRV) parameters as a measure of stress and recovery (VAS, visual analogue scale; STAI, state trait anxiety inventory).

RMSSD, is a measure of HRV that reflects parasympathetic nervous system activity. A meta-analysis of three studies (*n* = 43 participants) showed a statistically significant difference in RMSSD between stress and recovery periods (Hedges *g *=* −*0.63, *P *= 0.005, 95% CI: −1.07, −0.19) (Figure 2). The heterogeneity was low (*I*^2^ = 0%, might not be important).

**Figure 3.**
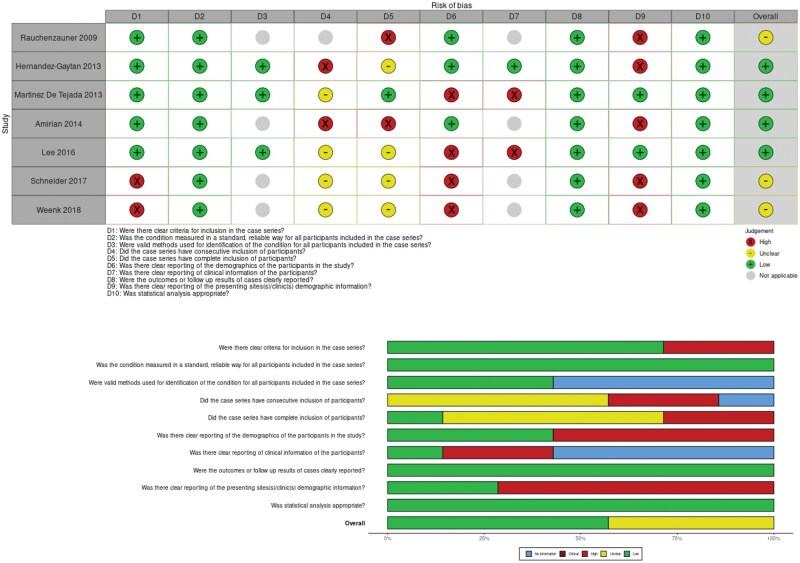
Risk of bias [28] of studies included in the meta-analysis.

SDNN, reflects both sympathetic and parasympathetic nervous system activity when measured over short periods; however, it is more reflective of sympathetic nervous system activity when measured for more than 24 hours. A meta-analysis of four studies (*n* = 63 participants) showed a statistically significant difference in SDNN between stress and recovery periods (Hedges *g *=* −*1.05, *P *= 0.001, 95% CI: −1.69, −0.41) (Figure 2). The heterogeneity was moderate (*I*^2^ = 64%, may represent substantial heterogeneity).

High frequency (HF), reflects parasympathetic nervous system activity. A meta-analysis of three studies (*n* = 43 participants) showed no statistically significant change in HRV between stress and recovery periods (Hedges *g *=* −*0.24, *P *= 0.28, 95% CI: −0.68, −0.20) (Figure 2). The heterogeneity was low (*I*^2^ = 5%, might not be important).

Low frequency (LF), reflects sympathetic nervous system activity. A meta-analysis of three studies (*n* = 43) showed a statistically significant change in HRV between the stress and recovery groups (Hedges *g *= 0.54, *P *= 0.01, 95% CI: 0.11, 0.97) (Figure 2). The heterogeneity was low (*I*^2^ = 0%, might not be important).

LF/HF ratio, reflects both sympathetic and parasympathetic nervous system function. This is based on LF being predominantly generated by the sympathetic nervous system and HF by the parasympathetic nervous system. Higher LF/HF ratios are reflective of sympathetic dominance and therefore an imbalance between sympathetic and parasympathetic nervous system activity [27]. Meta-analysis of two studies (*n* = 33) showed a statistically significant change in LF/HF ratio parameter between the stress and recovery group (Hedges *g *= 0.69, *P *= 0.006, 95% CI: 0.19, 1.19) (Figure 2). The heterogeneity was low (I^2^ = 0%, might not be important).

Two studies were not included in any of the meta-analyses. One study reported only LF power and HF power with *P* values but no means and standard deviations [21] and one study only reported mean values with no standard deviations [25] therefore it was not possible to include these data in the meta-analyses.

Study quality was assessed using the JBI checklist for case series [16]. This was converted into a risk of bias visualisation tool using Robvis (Figure 3) [29]. All studies had a high risk of bias; however, overall risk of bias was low or unclear for the studies included in the meta-analyses. The domains ‘D6—Was there clear reporting of the demographics of the participants in the study?’ and ‘D9—Was there clear reporting of the presenting sites demographic information?’ both presented the highest risk of bias.

The measuring and reporting of HRV data were assessed using standard for reporting diagnostic accuracy in studies using HRV (STARD_HRV_) [15]. Out of 25 points on the STARD_HRV_ scale, the number of points completed in each study ranged from 9.5 to 14.5 (see Table 3). The maximum completion rate against the STARD_HRV_ criteria was 48% with one study as low as 24%.

**Table 3.** Points completed against STARD_HRV_ criteria and reporting gaps (%)

Lead author	Year	STARD_HRV_ score (25)	Fully reported (%)	Partially reported (%)	Gaps in reporting (%)
Rauchenzauner	2009	11.0	24	32	44
Hernandez-Gaytan	2013	14.5	36	28	36
Martinez de Tejada	2013	10.0	32	28	40
Amirian	2014	14.5	36	24	40
Lee	2016	12.5	28	32	40
Schneider	2017	11.5	40	36	24
Weenk	2018	12.0	48	20	32

No studies were excluded on the grounds of heterogeneity. Heterogeneity ranged from 0% in the RMSSD, LF and LF/HF ratio sub-groups to 5% in the HF sub-group and 64% in the SDNN sub-group.

Sensitivity analysis was considered, however due to there being no clear outliers in terms of study quality this was not deemed to be appropriate. Additionally, it was not possible to assess publication bias due to the small number of studies included in this review.

## DISCUSSION

The main outcome of the review was that the individual HRV parameters SDNN, RMSSD, LF and LF/HF ratio all demonstrated a statistically significant change, and in the expected direction, between stressful and recovery (i.e. less stressful) periods as defined by the contextual or psychological measures. The HF parameter showed no statistically significant change between stress and recovery states.

SDNN is known to reflect both sympathetic and parasympathetic nervous system function [27]. The larger effect size in the SDNN sub-group may indicate that this parameter is more useful in detecting changes between stress and recovery states. This is likely due to both the sympathetic and parasympathetic relationship with SDNN in particular and that it is known to be more accurate when calculated over a 24-hour period which all studies included in this analysis were. Jastrow *et al*. [19], Lee *et al*. [26], and Schneider *et al*. [22] report both SNDD and RMSSD, and the effect size in the RMSSD group is far lower. This may be because RMSSD is known to be a better indicator of sympathetic nervous system function and therefore only stress states, so when used to detect changes between the two states the effect size is lower. Similarly, this may be the case in the LF/HF ratio sub-group.

The LF/HF ratio parameter is also known to detect both sympathetic and parasympathetic nervous system function, both stress and recovery, which again may be why it demonstrates a higher effect size. LF/HF ratio has previously been known as a marker of ‘sympatho-vagal balance’ [29]. This is a title that been challenged on the grounds that up to half of the variability may actually be due to parasympathetic activity [29,30], may be confounded by respiration and can vary greatly depending on testing conditions [31]. From this review, it could be postulated that SDNN may be more useful in detecting changes between stress and recovery states, but that RMSSD is better are detecting acute stress during real-time monitoring, hence its popularity in wearable technology. This would be useful if analysing HRV over longer time periods that is, a working week, whereby RMSSD could be used to detect acute changes in stress levels and SDNN used for tracking patterns of stress and recovery over the course of the week.

HF HRV is known to better reflect only parasympathetic activity, so while it may have utility for detecting recovery states, it may not be appropriate to use to detect changes between stress and recovery, this is supported with our findings that there was no statistically significant change between stress and recovery states for this parameter.

In their review of the metrics and norms that can be applied to HRV analysis, Chand *et al*. [32] identified the several independent variables that result in differences in HRV without any intervention, such as, age, gender, health status and physical fitness. This makes it important to adequately report participant demographics in conjunction with HRV, which was found to be lacking in several studies [19,22,23,33] when we conducted our risk of bias assessment. This may have occurred for a number of reasons, such as failing to recognise the need for within person analysis, failure to recognise that HRV is affected by so many individual variations, ethical or publication restrictions.

Perhaps most problematic is the variation in reporting of all of the HRV parameters. Our quality assessment against STARD_HRV_, showed demonstrable gaps in the reporting of HRV and our risk of bias assessment showed a particularly high risk of bias in both the reporting of both participant site demographics. We can also speculate that although HRV may seem like a simple objective measure of stress, if established guidelines are followed, such as the European Task Force [34] study methods as well as the analysis of HRV, becomes incredibly complex and may benefit from specialist electrophysiology support. Some publication bias arising from reporting only select parameters, which may arise for a number of reasons, such as aforementioned complexity in analysis or word count restrictions may mean that we are only evaluating/identifying work that has shown statistically significant results. We were unable to confirm this using funnel plots due to the small number of included studies in the review that met our stringent criteria.

Strengths of this review are that it was conducted according to the PRISMA guidelines [14] for conducting systematic reviews with the protocol registered with PROSPERO [13] in advance. It includes seven independent, international studies looking at HRV over stress and recovery periods in doctors. We were able to conduct meta-analysis for individual HRV parameters, RMSSD, SDNN, HF, LF and LF/HF.

Limitations are that the maximum number of participants included in meta-analysis was 63 and the lowest 43. Heterogeneity was high for the SDNN parameter and there was significant methodological variation across the studies. For example, with there being different medical specialities included, this meant there were different study environments with different definitions of being in high-stress, low-stress, or recovery environments. This is unavoidable in studies of doctors in their normal working environments in various locations internationally. Additionally, there are insufficient studies conducted in just any one speciality to do sub-group analysis. There was also a different HRV measurement device used in every study, in addition to the different methods to contextually or psychologically report stress. Notably, no included studies were conducted in the UK.

The studies included in this review were conducted between 2009 and 2018. Wearable technology has progressed since this time. Devices with longer battery life are now available, there are less invasive, more comfortable devices and advanced analytical software now available. While commercial devices offering HRV measurements are often worn on the wrist as a watch, with some now also offering ring based measurements. Due to the peripherally worn nature of these devices they are measuring pulse rate variability, often via photoplethysmography. This may present some additional challenges. First, vascular stiffening associated with age may affect the reliability of results [33]. Second, photoplethysmography measurements are known to be affected by skin tone [34] and finally, somewhat unique to this study population, is infection prevention control regulations that prevent both wrist watches or rings being worn by doctors in the workplace. Therefore, high quality HRV measurements in doctors may be best taken using chest worn devices capturing raw inter-beat interval data from ECG measurements.

The studies included in this review ranged from 24 to 48 hours in duration. Chronic stress and recovery imbalances contributing to burnout are likely to accumulate over much longer periods of time. Therefore, while this review indicates that HRV has some promise in detecting changes between stress and recovery states, utilizing the technology now available to us to take longer measurements may help us better understand the long-term patterns of occupational stress that contribute to burnout. Longer measurements would also then allow us to capture both occupational and life stressors as well as the behaviours and coping mechanisms utilised by individuals to deal with these stressors.

Both continuous measurement of HRV and completion of self-report measurements may introduce a degree of measurement reactivity during ambulatory assessment [35]. Future studies could aim to address this problem by ensuring that there is no immediate bio-feedback available to participants thus preventing behaviour modification secondary to visualisation of their own data. Furthermore, any self-report measures could be repeated at over the course of a study period. This could also be completed utilising technology available to fill in surveys remotely via mobile phone for example.

In a post-pandemic era where the challenges within our medical workforces are unprecedented, with the vast majority of individuals reporting moderate to high levels of burnout in the most challenged specialties, there is an urgent requirement for further high-quality studies utilising up to date HRV technology as a biomarker of stress to enhance our understanding of stress and recovery in medical doctors. These studies should have robust basic methodology and adhere to the European Task Force for HRV studies [34] and report in line with STARD_HRV_ [15]. Greater understanding of how individual HRV parameters can be utilised as health metrics in this context is required.

This review finds that the HRV parameters RMSSD, SDMM, LF and LF/HF ration can detect a statistically significant change between stress and recovery states in medical doctors in their usual clinical workplaces. Due to relatively small study sample sizes, methodological heterogeneity in the included studies and moderate study quality, it is not possible to draw any meaningful clinical conclusions. Little is known about what the minimum meaningful clinical difference in HRV would need to be between stress and recovery to positively reduce burnout risk. Therefore, we would recommend robust measurement techniques and comprehensive reporting of HRV parameters in keeping with the European Task Force [34] and STARD_HRV_ [15] as well as strong basic study methodology, particularly in reporting of participant demographics and within person design for future studies. This may help inform the clinical application for HRV as a biomarker of occupational stress.

## Supplementary Material

kqaf101_Supplementary_Data

## Data Availability

Data are available on request.

## ABBREVIATIONS

ANS Autonomic nervous systemECG ElectrocardiogramGMC General Medical CouncilHRV Heart rate variabilityJBI Joanna Briggs InstitutePNS Parasympathetic nervous systemRevMan Review managerSD Standard deviationSNS Sympathetic nervous systemSTARDHRV Standard for reporting diagnostic accuracy in heart rate variability

## ABBREVIATIONS:

ANS: Autonomic nervous system
ECG: Electrocardiogram
GMC: General Medical Council
HRV: Heart rate variability
JBI: Joanna Briggs Institute
PNS: Parasympathetic nervous system
RevMan: Review manager
SD: Standard deviation
SNS: Sympathetic nervous system
STARD_HRV_: Standard for reporting diagnostic accuracy in heart rate variability

## References

[1] General Medical Council. *Burnout for Trainees and Trainers*, 2022. https://reports.gmc-uk.org/analytics/saw.dll?Dashboard&PortalPath=%2Fshared%2FEducation%20Reports%2FEvidence%20Reports%2FBurnout%20Reports%2FBurnout-Dashboard-Public&Page=All%20doctors%20UK-wide&P1=dashboard&Action=Navigate&ViewState=veo76pusm0vs5ea1mpjt7lcbi2&P16=NavRuleDefault&NavFromViewID=d%3Adashboard∼p%3Aevkf7qvbniab2isj (23 October 2023, date last accessed).

[2] O’Callaghan J , Mohan HM, Sharrock A et al Cross-sectional study of the financial cost of training to the surgical trainee in the UK and Ireland. BMJ Open 2017;7:e018086. 10.1136/bmjopen-2017-018086 PMC5695344 29146646

[3] International Classification of Diseases. *QD85 Burnout*, 2021. https://icd.who.int/browse11/l-m/en#/http://id.who.int/icd/entity/129180281 (23 September 2021, date last accessed).

[4] Bakker AB , Demerouti E. The job demands‐resources model: state of the art. J Manage Psychol 2007;22:309–328.

[5] Cottey L , Roberts T, Graham B et al Need for recovery and physician well-being in emergency departments: national survey findings. Eur J Emerg Med. 10.1097/MEJ.0000000000000850 34285172

[6] Shaffer FB , Moss D. Review of I. Z. Khazan (2019), biofeedback and mindfulness in everyday life: practical solutions for improving your health and performance. W. W. Norton and company. Appl Psychophysiol Biofeedback 2022;47:357–360. 36269523 10.1007/s10484-022-09564-0

[7] Altini M. *Data Interpretation Issues in Wearables*. https://www.marcoaltini.com/blog (3 August 2024, date last accessed).

[8] Thielmann B , Pohl R, Böckelmann I. Heart rate variability as a strain indicator for psychological stress for emergency physicians during work and alert intervention: a systematic review. J Occup Med Toxicol 2021;16:24. 34187497 10.1186/s12995-021-00313-3 PMC8240085

[9] Thielmann B , Karlsen H, Darius S, Bockelmann I. Heart rate variability in different levels of burnout-cross-sectional study of different occupational groups heart rate variability and burnout. J Occup Environ Med 2021;63:e622–e630. 34173785 10.1097/JOM.0000000000002307

[10] Russell G , Lightman S. The human stress response. Nat Rev Endocrinol 2019;15:525–534. 31249398 10.1038/s41574-019-0228-0

[11] Menon NK , Shanafelt TD, Sinsky CA et al Association of physician burnout with suicidal ideation and medical errors. JAMA Netw Open 2020;3:e2028780. 33295977 10.1001/jamanetworkopen.2020.28780 PMC7726631

[12] Trockel M , Bohman B, Lesure E et al A brief instrument to assess both burnout and professional fulfilment in physicians: reliability and validity, including correlation with self-reported medical errors, in a sample of resident and practicing physicians. Acad Psychiatry 2018;42:11–24. 29196982 10.1007/s40596-017-0849-3 PMC5794850

[13] Kane L , Martin K, Ball D, Powell D, Rees C, Curran J. *Can Continuous Heart Rate Variability Monitoring, Combined With Contextual and Psychological Measures be Used to Detect Patterns of Stress and Recovery in Medical Doctors?* https://www.crd.york.ac.uk/PROSPERO/display_record.php?RecordID=413282 (1 September 2024, date last accessed).

[14] Higgins JPT , Thomas J, Chandler J, Cumpston M, Li T, Page MJ, Welch VA. *Cochrane Handbook for Systematic Reviews of Intervention*, 2023. www.training.cochrane.org/handbook (27 February 2024, date last accessed).

[15] Dobbs WC , Fedewa MV, MacDonald HV et al The accuracy of acquiring heart rate variability from portable devices: a systematic review and meta-analysis. Sports Med 2019;49:417–435. 30706234 10.1007/s40279-019-01061-5

[16] Munn Z , Barker TH, Moola S et al Methodological quality of case series studies: an introduction to the JBI critical appraisal tool. JBI Evid Synth 2020;18:2127–2133. 33038125 10.11124/JBISRIR-D-19-00099

[17] Review Manager, Version 5. 2022 The Cochrane Collaboration. Available at revman.cochrane.org

[18] Porta M. In A Dictionary of Epidemiology : Oxford University Press. Retrieved 2 October 2025

[19] Jastrow N , Poncet A, Le Scouezec I, Irion O, Kayser B. Perceived and measured physical activity and mental stress levels in obstetricians. Eur J Obstet Gynecol Reprod Biol 2013;171:44–48. 23998555 10.1016/j.ejogrb.2013.08.020

[20] Lee H , Chen B, Lo S, Chen P, Guo Y. Predicting physician’s duty stress by parasympathetic nervous function (also to be considered for mini-symposium: early detection and management of workers under stress). Occup Environ Med. 2014;71:A93.

[21] Amirian I , Aring T, Andersen L, Rosenberg J, GÃgenur I. Decreased heart rate variability in surgeons during night shifts. Journal Canadian de Chirurgie, Can J Surg 2014;57:300. 10.1503/cjs.028813 PMC4183675 25265102

[22] Schneider F , Martin J, Hapfelmeier A, Jordan D, Schneider G, Schulz CM. The validity of linear and non-linear heart rate metrics as workload indicators of emergency physicians. PLoS One 2017;12:e0188635. 29190808 10.1371/journal.pone.0188635 PMC5708782

[23] Weenk M , Alken APB, Engelen L, Bredie SJH, van de Belt TH, van Goor H. Stress measurement in surgeons and residents using a smart patch. Am J Surg 2018;216:361–368. 28615137 10.1016/j.amjsurg.2017.05.015

[24] Hernandez-Gaytan S , Rothenberg S, Landsbergis P et al Job strain and heart rate variability in resident physicians within a general hospital. Am J Ind Med 2013;56:38–48. 22886873 10.1002/ajim.22098

[25] Rauchenzauner M , Ernst F, Hintringer F et al Arrhythmias and increased neuro-endocrine stress response during physicians’ night shifts: a randomized cross-over trial. Eur Heart J 2009;30:2606–2613. 19602503 10.1093/eurheartj/ehp268

[26] Lee H-H , Lo S-H, Chen B-Y et al Increased night duty loading of physicians caused elevated blood pressure and sympathetic tones in a dose-dependent manner. Int Arch Occup Environ Health 2016;89:413–423. 26231250 10.1007/s00420-015-1080-8

[27] Munn Z , Peters MDJ, Stern C, Tufanaru C, McArthur A, Aromataris E. Systematic review or scoping review? Guidance for authors when choosing between a systematic or scoping review approach. BMC Med Res Methodol 2018;18:143. 30453902 10.1186/s12874-018-0611-x PMC6245623

[28] McGuinness LA , Higgins JPT. *Risk-of-Bias VISualization (Robvis): An R Package and Shiny Web App for Visualizing Risk-of-Bias Assessments*. https://mcguinlu.shinyapps.io/robvis/ (27 February 2024, date last accessed). 10.1002/jrsm.1411 32336025

[29] Shaffer F , Ginsberg JP. An overview of heart rate variability metrics and norms. Front Public Health 2017;5:258. 29034226 10.3389/fpubh.2017.00258 PMC5624990

[30] Torkamani-Azar M , Lee A, Bednarik R. Methods and measures for mental stress assessment in surgery: a systematic review of 20 years of literature. IEEE J Biomed Health Inform 2022;26:4436–4449. 35696473 10.1109/JBHI.2022.3182869

[31] Task Force of the European Society of Cardiology the North American Society of Pacing Electrophysiology. Heart rate variability. Circulation 1996;93:1043–1065. 8598068

[32] Chand K , Chandra S, Dutt V. A comprehensive evaluation of linear and non-linear HRV parameters between paced breathing and stressful mental state. Heliyon 2024;10:e32195. 38873683 10.1016/j.heliyon.2024.e32195 PMC11170182

[33] Schiffrin EL. Vascular stiffening and arterial compliance: implications for systolic blood pressure. Am J Hypertens 2004;17:39S–48S. 15607434 10.1016/j.amjhyper.2004.08.019

[34] Kamal AAR , Harness JB, Irving G, Mearns AJ. Skin photoplethysmography–a review. Comput Methods Programs Biomed 1989;28:257–269. 2649304 10.1016/0169-2607(89)90159-4

[35] König LM , Allmeta A, Christlein N, Van Emmenis M, Sutton S. A systematic review and meta-analysis of studies of reactivity to digital in-the-moment measurement of health behaviour. Health Psychol Rev 2022;16:551–575. 35264084 10.1080/17437199.2022.2047096

