# Supplementary material for: Continuous heart rate variability monitoring, stress and recovery in doctors: a systematic review and meta-analysis
Source: Occup Med (Lond). 2025 Oct 28;75(9):630–9. doi: 10.1093/occmed/kqaf101 (PMC12794872; doi:10.1093/occmed/kqaf101)
Supplement: kqaf101_Supplementary_Data [file kqaf101_supplementary_data.doc]

**Appendix 1**

HRV is a complex biomarker, although often referred to simply as HRV there are in fact 37 commonly used parameters. These 37 parameters are generally described in two categories, linear and non- linear. Each of these parameters then can be utilised in different ways for different purposes reflecting different parts of autonomic nervous system function. A full description of heart rate variability metrics, their norms and associations is described by Shaffer and Ginsberg^(28)^ , additionally Chand et al provide a useful description of their utility in measuring stress and recovery.^(35)^A summary of parameters described in our review and their relevant associations can be found in **Table 4**.

| Parasympathetic Nervous System | Sympathetic Nervous System | Circadian |
| --- | --- | --- |
| SDNNI | SDNN | ULF |
| RMSSD | SDNNI | Spectral |
| LK | VLF |  |
| LF/HF | LF |  |
|  | HF |  |
|  | LF/HF |  |

**Table 4: A summary of heart rate variability (HRV) parameters and corresponding autonomic nervous system function relevant to stress and recovery measurements;**

**Appendix 2**

PRISMA checklist – attached as separate document.

**Appendix 3**

Searches were conducted in collaboration with a medical research librarian to develop a comprehensive search strategy. Medical Subject Headings (MeSH) and keywords detailed in Table 1 were used. We searched MEDLINE, Cochrane, Embase, PsychINFO. Reference lists of included papers will be searched for relevant citations. Grey literature will be searched in google scholar, MedArXiv and PsyArXiv. Abstracts will be reviewed against inclusion criteria and eligible full text papers will be retrieved.

Table 5.

|  | **AND** | **AND** |  |
| --- | --- | --- | --- |
| **OR** | **Population** | **Condition** | **Measurement** |
| **OR** | exp physicians/ | Occupational Stress/ | (heart rate adj3 variab$).tw. |
| **OR** | Doctor$.tw | Burnout/ | heart rate variability.tw,kw. |
| **OR** | clinician$.tw,kw. | Stress, Psychological/ | HRV |
| **OR** | GP.tw,kw. | Workload/ | Pulse rate variability.tw.kw |
| **OR** | General Practitioner$.tw,kw | burnout.tw. |  |
| **OR** | emergency physician$.tw,kw. | work?place stress$.tw,kw. | Autonomic nervous system |
| **OR** | emergency doctor$.tw,kw. | professional stress$.tw. | cardiac autonomic control.tw,kw. |
| **OR** | helicopter physician$.tw. | work related stress$.tw. | autonomic function$.tw,kw. |
| **OR** | helicopter doctor$.tw,kw. | job stress$.tw. | cardiac vagal tone$.tw,kw. |
| **OR** | doctor on call.tw,kw. | job related stress$.tw. | autonomic cardiac modulation$.tw,kw. |
| **OR** | doctor in training.tw,kw. | job strain$.tw. | Vagus Nerve/ |
| **OR** | foundation doctor$.tw,kw. |  | vagal tone$.tw,kw. |
| **OR** | foundation physician$.tw,kw. |  | vagal activit$.tw,kw. |
| **OR** | trainee doctor$.tw,kw. |  |  |
